# Increased COVID-19 mortality rate in rare disease patients: a retrospective cohort study in participants of the Genomics England 100,000 Genomes project

**DOI:** 10.1186/s13023-022-02312-x

**Published:** 2022-04-12

**Authors:** Huayu Zhang, Johan H. Thygesen, Ting Shi, Georgios V. Gkoutos, Harry Hemingway, Bruce Guthrie, Honghan Wu, Genomics England Research Consortium

**Affiliations:** 1 Advanced Care Research Centre, Usher Institute, University of Edinburgh, Edinburgh, UK; 2 Institute of Health Informatics, University College London, London, UK; 3 Centre for Global Health, Usher Institute, University of Edinburgh, Edinburgh, UK; 4 Institute of Cancer and Genomics, University of Birmingham, Birmingham, UK

**Keywords:** Rare diseases, COVID-19 mortality

## Abstract

**Background:**

Several common conditions have been widely recognised as risk factors for COVID-19 related death, but risks borne by people with rare diseases are largely unknown. Therefore, we aim to estimate the difference of risk for people with rare diseases comparing to the unaffected.

**Method:**

To estimate the correlation between rare diseases and COVID-19 related death, we performed a retrospective cohort study in Genomics England 100k Genomes participants, who tested positive for Sars-Cov-2 during the first wave (16-03-2020 until 31-July-2020) of COVID-19 pandemic in the UK (n = 283). COVID-19 related mortality rates were calculated in two groups: rare disease patients (n = 158) and unaffected relatives (n = 125). Fisher’s exact test and logistic regression was used for univariable and multivariable analysis, respectively.

**Results:**

People with rare diseases had increased risk of COVID19-related deaths compared to the unaffected relatives (OR [95% CI] = 3.47 [1.21– 12.2]). Although, the effect was insignificant after adjusting for age and number of comorbidities (OR [95% CI] = 1.94 [0.65–5.80]). Neurology and neurodevelopmental diseases was significantly associated with COVID19-related death in both univariable (OR [95% CI] = 4.07 [1.61–10.38]) and multivariable analysis (OR [95% CI] = 4.22 [1.60–11.08]).

**Conclusions:**

Our results showed that rare disease patients, especially ones affected by neurology and neurodevelopmental disorders, in the Genomics England cohort had increased risk of COVID-19 related death during the first wave of the pandemic in UK. The high risk is likely associated with rare diseases themselves, while we cannot rule out possible mediators due to the small sample size. We would like to raise the awareness that rare disease patients may face increased risk for COVID-19 related death. Proper considerations for rare disease patients should be taken when relevant policies (e.g., returning to workplace) are made.

**Supplementary Information:**

The online version contains supplementary material available at 10.1186/s13023-022-02312-x.

## Introduction

The ongoing SARS-CoV-2 pandemic has resulted in more than 307 million cases and 5.49 million deaths worldwide [1] by 08-Sep-2021. There is considerable policy, clinical and public interest in who should be prioritised for vaccination, in the face of uncertainty about the benefits and risks of COVID-19 vaccines, and concerns about the ongoing limited supplies of vaccines in many areas and countries. Several common pre-existing medical conditions have been identified as risk factors for severe COVID-19 [2, 3]. However, the risk of severe COVID-19 in people with rare diseases is uncertain. Although individually rare, rare diseases are cumulatively common, affecting approximately 1 in 17 people in the UK, which means over 3.5 million people are affected [4]. They can be both life-limiting and life-threatening, resulting in a substantial impact on the education, financial status, mobility and mental health. Therefore, it is important to consider rare disease patients when developing relevant policy. Better knowledge regarding risk factors for severe COVID-19 could help guide decisions on mitigating exposure, inform risk management and in targeting vaccines to those most at risk [5].

Studies have been done on the indirect influence of SARS-CoV-2 pandemic on people with rare diseases, including patient’s health status (non-COVID-19 related), health service use patterns, mental health, daily living, social life, and financial status [6–8].

Understanding and directly measuring the risk of COVID-19 related mortality in people with rare diseases is important but difficult due to the relatively small size of rare disease specific cohorts and poor coding of some rare diseases in larger scale health records. To date, direct analysis on the risk of COVID-19 mortality among people with rare diseases is still limited. In a Hong Kong study by Chung et al. increased COVID-19 related mortality was observed in hospitalised patients with rare diseases compared to the general population, but other COVID-19 related commodities were not accounted for [9]. To address the above challenge, we utilised data in the Genomics England 100k Genomes project, which has a specific focus on recruiting rare-disease patients with clinical diagnosis available [10].

The current study sought to understand the direct impact of existing rare disease on COVID-19-related mortality rate. We adjusted for age and COVID-19 related comorbidities in the multivariable analysis, since these are known risk factors for COVID-19 related death [3].

## Methods

### Ethical approval

The study was approved by Genomics England under “Approval of GeCIP Project 450”.

### Study design and setting

We performed a retrospective cohort study design to assess the difference in COVID-19 associated mortality rate (outcome) between people with rare diseases and without rare diseases (unaffected relatives) from the participants of the Genomics England 100k Genomes project tested positive for COVID-19. Our study question is whether pre-existing rare diseases independently increase the risk of COVID-19 related deaths.

### Participants and recruitment criteria

**Fig. 1:**
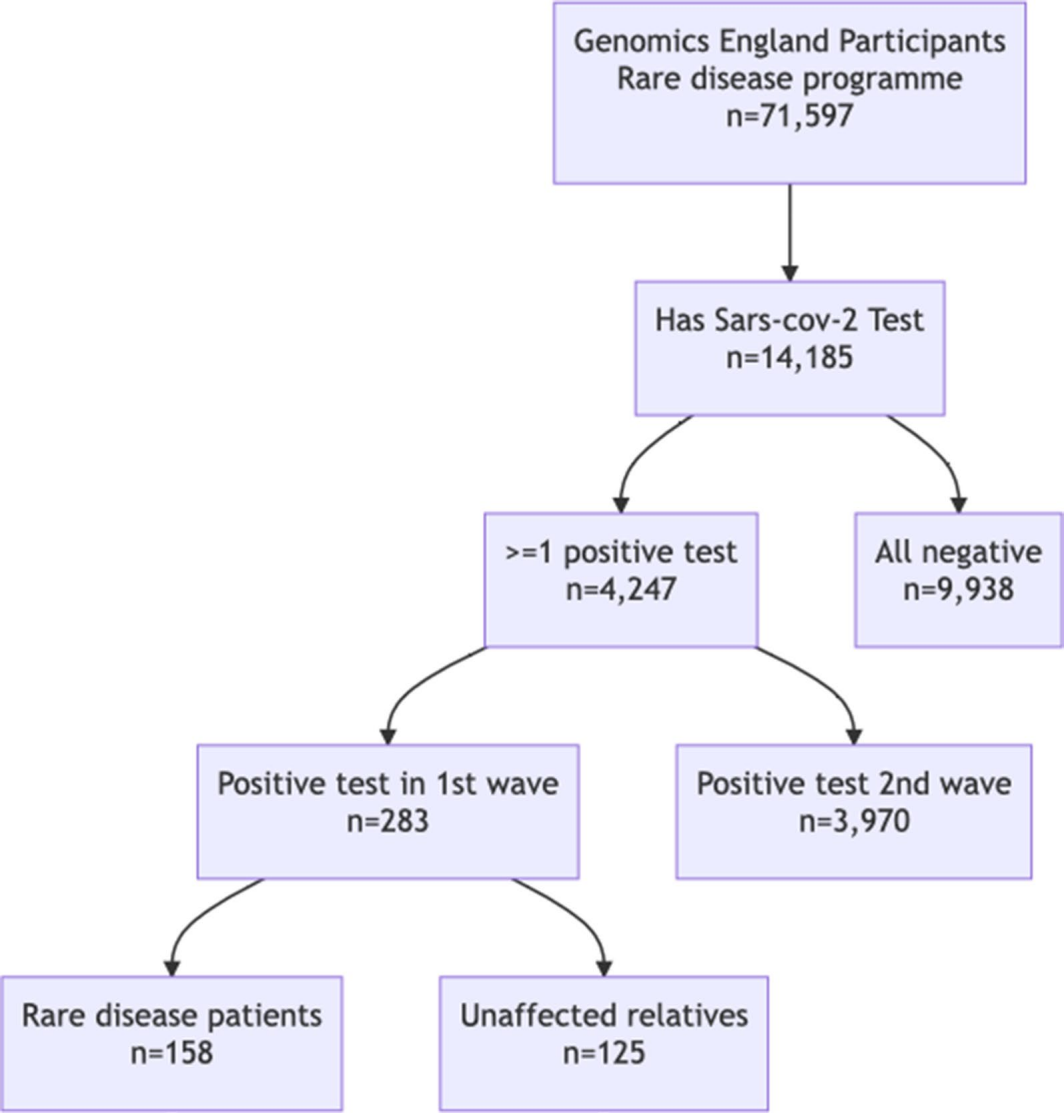
Diagram for cohort participant selection

Participants with at least one of 190 different rare diseases and relatives were recruited to the Genomics England 100k Genomes project, where participants had a provisional diagnosis instead of a molecular diagnosis. In addition, there were unaffected relatives who were invited to participate when rare disease participants (probands) were recruited to the Genomics England 100k Genomes project. Full recruitment criteria for the Genomics England 100k Genomes project can be found at link (Please see the full link in footnote^1^). The study cohort consists of 283 participants with at least one positive test during the first wave of pandemic in the UK (from 16-03-2020 to 31-07-2020) (Fig. 1). Individuals in the cohort were followed up until 30-09-2020. The cohort was further divided by two groups, rare disease participants and unaffected relatives, based on whether the participant was affected by at least one rare disease (Fig. 1).

### Variables and data source

Death records and associated underlying reasons were obtained from Office of National Statistics (ONS) until 30-09-2020. Records with International Statistical Classification of Diseases (ICD-10) codes of U07.1 or U07.2 in any fields of causes of death were defined as *COVID-19-related death*, although there were no records of ICD-10:U07.2 in the cohort. The rare disease condition (disease groups and specific diseases) was retrieved from the Genomics England 100k Genomes project. A binary variable *Affected by rare diseases* was derived indicating if an individual was affected by rare diseases.

We also included confounders such as demographic variables and common risk factors for COVID-19. They were selected according to the risk stratification research done by International Severe Acute Respiratory and emerging Infection Consortium (ISARIC) [3]. Age was defined as the age by year on the day of first positive test and was converted to a binary variable *Age* ($\ge 60$). COVID-19 related risk factors (chronic cardiovascular disease, chronic renal disease, malignant neoplasm, moderate to severe liver disease, diabetes mellitus, clinician-defined obesity and chronic respiratory disease) were included as potential mediators of effects based on the ISARIC4C risk prediction model [3]. International Statistical Classification of Diseases 10 (ICD-10) codes corresponding to these conditions were obtained from the HDR UK Phenotype Library [11] (Additional file 1: Table S2). Participant level diagnosis (also in ICD-10) was extracted and curated from Admitted Patient Care (HES-APC) and Outpatients (HES-OP) data of Hospital Episode Statistics (HES). We counted how many comorbidities each person had which had been identified by ISARIC as increasing the risk of COVID-19 serious disease or death. Numbers of comorbidities were calculated as the counts of total comorbidities and were used to derive the binary variable *Number of comorbidities* $\ge 2$.

### Statistical analyses

Clinical characteristics were reported as count(percentage in group) and median[interquartile range] for binary and continuous variables, respectively. Difference of clinical characteristics between individuals affected by rare diseases and unaffected relatives were determined by Fisher’s exact test and Student’s t-test for binary and continuous variables, respectively. P-values were reported for variables that were significantly different between groups. Univariable analysis on the associations between *Affected by rare diseases* and *COVID-19 related death* was carried out using Fisher’s exact test. Univariable odds ratios (uOR) and their corresponding 95% Confidence Interval (95% CI) were reported. In case of zero outcome frequency in one group, we reported the frequencies directly. Adjusted odds ratios were calculated with multivariable logistic regression model. Multivariable odds ratios (mOR) with 95% CI were reported. Multivariable ORs for *Age* ($\ge 60$) and *Number of comorbidities* $\ge 2$ were calculated in logistic regression analysis with *Age* ($\ge 60$), *Number of comorbidities* $\ge 2$ and *Affected by rare diseases* as independent variables. For calculation of multivariable ORs for the variables of interest (*Affected by rare diseases* or individual rare diseases), *Age* ($\ge 60$) and *Number of comorbidities* $\ge 2$ were adjusted. Multivariable ORs of each of the variables of interest were calculated in individual logistic regression analyses.

## Results

Our cohort included 283 participants from the 71,597 participants in the rare disease programme of the Genomics England 100k Genomes project, who tested positive for SARS-CoV-2 in the first wave of COVID-19 pandemic in the UK (defined by 16-03-2020 to 31-July-2020). Baseline clinical characteristics were illustrated in Table 1. The fraction of participants with age $\ge$ 60 was larger in rare disease participants. Proportion of male participants was higher in rare disease participants. In addition, frequencies of chronic cardiovascular disease, chronic kidney disease, malignant neoplasm and chronic pulmonary disease were statistically significantly higher in rare disease participants. Numbers of COVID-19 related comorbidities was higher in rare disease participants. For existing rare diseases, the most prevalent groups were neurology and neurodevelopment disorders, renal and urinary tract disorders, cardiovascular disorders and ophthalmological disorders.

**Table 1:** Clinical characteristics

	All (n = 283)	With rare disease (n = 158)	Unaffected relatives (n = 125)	
Demography				
Age (years)	45.0 [36.0–58.0]	49.5 [33.5–62.0]	42.0 [37.0–57.0]	–
Age ($\ge$60)*	64 (22.6%)	48 (30.4%)	16 (12.8%)	$p<0.001$
Sex (male)*	128 (45.2%)	80 (50.6%)	48 (38.4%)	$p=0.042$
Comorbidities				
Chronic cardiovascular disease*	55 (19.4%)	46 (29.1%)	9 (7.2%)	$p<0.001$
Chronic kidney disease*	41 (14.5%)	36 (22.8%)	$\le$5 ($\le$4.0%)#	$p<0.001$
Malignant neoplasm*	30 (10.6%)	23 (14.6%)	7 (5.6%)	$p=0.019$
Moderate or severe liver disease	7 (2.5%)	$\le$5 ($\le$3.2%)	$\le$5 ($\le$4.0%)	–
Obesity (Clinician defined)	62 (21.9%)	35 (22.2%)	27 (21.6%)	–
Chronic pulmonary disease*	49 (17.3%)	35 (22.2%)	14 (11.2%)	$p=0.018$
Diabetes (Type 1 and 2)	33 (11.7%)	19 (12.0%)	14 (11.2%)	–
Number of COVID-19 related comorbidities ($\ge$2)*	79 (27.9%)	60 (38.0%)	19 (15.2%)	$p<0.001$
Rare disease groups				
Neurology and neurodevelopmental disorders	66 (23.3%)	66 (41.8%)	–	–
Renal and urinary tract disorders	26 (9.2%)	26 (16.5%)	–	–
Cardiovascular disorders	20 (7.1%)	20 (12.7%)	–	–
Ophthalmological disorders	12 (4.2%)	12 (7.6%)	–	–
Other groups	34 (12.0%)	34 (21.5%)	–	–
Rare disease—Specific diseases				
Epilepsy plus other features	18 (6.4%)	18 (11.4%)	–	–
Cystic kidney disease	11 (3.9%)	11 (7.0%)	–	–
Intellectual disability	10 (3.5%)	10 (6.3%)	–	–
Hereditary ataxia	10 (3.5%)	10 (6.3%)	–	–
Unexplained kidney failure in young people	6 (2.1%)	6 (3.8%)	–	–
Ultra-rare undescribed monogenic disorders	6 (2.1%)	6 (3.8%)	–	–
Rod-cone dystrophy	6 (2.1%)	6 (3.8%)	–	–
Other diseases	23 (14.6%)	20 (12.7%)	–	–

Clinical characteristics were reported as count (percentage in group) and median[interquartile range] for binary and continuous variables, respectively.

*Statistically significant difference in the comparison between rare disease patients and unaffected relatives

#Frequencies less than 5 are suppressed due to requirement of data governance

In a univariable analysis, rare disease condition was strongly associated with death from COVID-19 (univariable odds ratio (uOR) = 3.47, 95% CI 1.21–12.2) (Table 1). After adjustment for *Age* ($\ge 60$ *years*) and COVID-19 related comorbidities (*Number of commorbidities* $\ge 2$), the estimated multivariable odds ratio (mOR) of death in people with rare diseases was 1.94, although this was not statistically significant (95% CI 0.65–5.48) (Table 2).

**Table 2:** Odds ratio of COVID-19 mortality risk factors in univariable and multivariable analyses

	Univariable OR [95% CI]	Multivariable OR [95% CI]
Age ($\ge$60)	14.78 [5.31–47.86]	9.95 [3.52–28.17]*
No of comorbidities ($\ge$2)	5.46 [2.15–14.77]	2.10 [0.79–5.58]**
Affected by rare diseases	3.47 [1.21–12.18]	1.94 [0.65–5.80]#
Neurology and neurodevelopmental disorders	4.07 [1.61–10.38]	4.22 [1.60–11.08]#
Early onset dystonia	5.28 [0.09–104.83]	26.64 [2.01–352.67]#
Early onset and familial Parkinson’s Disease	10.92 [0.76–157.41]	11.99 [1.25–114.71]#
Intellectual disability	2.70 [0.26–14.71]	8.10 [1.11–59.00]#

*Adjusted by *Number of comorbidities* $\ge 2$ and *Affected by rare diseases*

**Adjusted by *Age* ($\ge 60$) and *Affected by rare diseases*

#Adjusted by *Age* ($\ge 60$) and *Number of comorbidities* $\ge 2$ in individual logistic regression analysis

Neurology and neurodevelopmental disorders were significantly associated with COVID19-related death in both univariable (uOR = 4.07, 95% CI 1.61–10.38) and multivariable analysis (mOR = 4.22, 95% CI 1.60–11.08]). Further analysis with specific rare diseases revealed that Early onset dystonia (mOR = 26.64, 95% CI 2.01–352.67), Early onset and familial Parkinson’s Disease (mOR = 11.99, 95% CI 1.25–114.71) and Intellectual disability were significantly associated with (mOR = 8.10, 95% CI 1.11–59.00), all of which belong to neurology and neurodevelopment disorders. Odds ratios of all analyses with rare disease groups and specific disease can be found in Additional file 2: Table S1.

## Discussion

Our results showed that rare disease participants who tested positive for SARS-CoV-2 in the Genomics England 100k Genomes projects had increased risk of COVID-19 related death compared to their unaffected relatives during the first wave of the pandemic in UK, although the increase was not significant after accounting for age and number of COVID-19 related comorbidities. This is probably because rare disease patients had significantly higher frequencies of certain comorbidities and a higher number of comorbidities, which is known to affect the COVID-19 related mortality. Moreover, the sample size of this study limits our ability to establish the significant association. Majority of the increase was attributed to neurology and neurodevelopment disorders, which was significantly associated with COVID19 related death in both univariable and multivariable analysis.

Our results are in line with early reports by the Hong Kong study done by Chung et al., in which increased COVID-19-related hospital mortality was observed (mOR = 3.4, 95% CI 1.24–9.41) in rare disease patients compared with the general population, after adjusting for admission age [9]. There are two differences in the settings in our study: (1) our cohort includes individuals with positive Sars-CoV-2 tests, while the cohort of the Hong Kong study only considered hospitalised patients. (2) The Hong Kong study did not account for COVID-19 related comorbidities in the multivariable analysis, many of which are commonly found in rare disease patients and affecting the COVID-19 related mortality. In addition, our study had a slightly larger sample size for people with rare diseases (125 in our study compared to 77 in Hong Kong study).

Our study has contributed to UK evidence using specialised rare disease patient cohort and quantified the strength of associations between rare diseases and COVID-19 related mortality accounting for age and COVID-19 related comorbidities. Current UK guideline for vulnerable groups for COVID-19 includes “conditions affecting the brain or nerves”. Our observation on the increased COVID19 related death in people with neurology and neurodevelopmental disorders adds evidence to the above term in the context of rare diseases. This will help inform risk management decisions and in targeting vaccines. With booster vaccines being administered or planned internationally and nationally, policy makers will be able to use data from our study to guide decisions on booster vaccination priorities among rare disease patients, together with other public health surveillance data.

There were several limitations of our study. First, the population size is small which could result in inaccurate estimation of contribution of different factors (as reflected in the wide confidence intervals of our odds ratios). Also, the small sample size did not allow us to carry out thorough subgroup analysis or draw conclusions on one single rare disease. Second, mortality rate estimation could be biased by different healthcare requirements, concerning the testing strategy in the 1st wave of pandemic (high risk patients had better chance to get tested). If there were more cases with mild COVID-19 who were not tested in one group, the mortality rate in that group would be over-estimated. These limitations will be addressed in cohorts with larger sample size.

In conclusion, participants with rare diseases (especially ones with neurology and neurodevelopmental diseases) in the Genomics England 100k Genomes project had increased risks of COVID-19 related mortality during the first wave of the pandemic in UK, but the findings should be interpreted cautiously as the sample size is small. Further work is needed to replicate the findings in larger datasets and to better account for confounders and mediators. Existing rules for defining those who are clinically vulnerable to inform shielding decisions and vaccination prioritisation may therefore fail to identify people at risk of serious COVID-19 due to rare diseases. Based on the findings, we advocate for tailored protections for people with rare diseases (e.g., prioritised (booster) vaccination scheduling and personalised policy for returning to work).

## Supplementary information

**Additional file 1: Table S2.** Lists of ICD-10 codes for comorbidities associated to COVID-19

**Additional file 2: Table S1.** Univariable and multivariable ORs for association between rare disease groups/specific diseases and COVID-19

## Data Availability

De-identified individual participant data underlie the results reported in this article can be shared together with the study protocol, statistical analysis plan and analytic code, immediately following publication and ending 2 years following article publication. Data will be shared with investigators whose proposed use of the data has been approved by an independent review committee identified for individual participant data meta-analysis. Proposals may be submitted up to 2 years following article publication to Genomics England data governance team. Information regarding submitting proposals and accessing data may be found at .

## Footnotes

1 https://www.GenomicsEngland.co.uk/about-genomics-england/the-100000-genomes-project/information-for-gmc-staff/rare-disease-documents/rare-disease-eligibility-criteria/
